# Properties of Concrete Containing Recycled Glass Aggregates Produced of Exploded Lighting Materials

**DOI:** 10.3390/ma13010226

**Published:** 2020-01-04

**Authors:** Tomasz Drzymała, Bartosz Zegardło, Piotr Tofilo

**Affiliations:** 1 The Main School of Fire Service, Faculty of Safety Engineering and Civil Protection, ul. Słowackiego 52/54, 01-629 Warsaw, Poland; tdrzymala@sgsp.edu.pl; 2 Faculty of Natural Sciences, Siedlce University of Natural Sciences and Humanities, ul. Konarskiego 2, 08-110 Siedlce, Poland; bart.z@wp.pl

**Keywords:** glass aggregates, glass waste, recycled concrete, lighting glass

## Abstract

The paper presents an analysis of the possibilities of using glass waste from recycled lighting materials as aggregates for cement concrete. The research material was obtained from a company that utilizes electrical waste. Glass from pre-sorted elements was transported to the laboratory and crushed in a drum crusher. In this way, the aggregate obtained was subjected to the basic tests that are carried out for aggregates traditionally used in construction. The specific density of aggregate, bulk density, absorbability, crushing index, grain shape, texture type and aggregate flatness index were examined. In the next stage of research work, concrete mixtures were made in which crushed aggregate from crushed fluorescent lamps was used as a substitute for gravel aggregate. Mixtures containing 10%, 30%, 50% and 100% aggregate were made. A mixture containing only sand and gravel aggregate was made as a comparative mixture. Basic tests of both fresh concrete mix and hardened concrete were carried out for all concrete made. The consistency of the fresh concrete mix, the air content in the concrete mix, the density of hardened concrete, absorbability, water permeability under pressure and the basic compressive and tensile (flexular) strength tests were performed. The test results showed that the greater the addition of recycled glass aggregate, the less advantageous are the features of the concrete obtained with its participation. Microscopic analyses carried out in order to explain this phenomenon indicated an unfavorable influence of the grain shape of the aggregate thus obtained. Despite this fact, recycling of lighting waste in concrete composites is recommended as a pro-ecology measure; however, attention was paid to the benefits of using only 30% by mass of said waste in relation to the weight of the traditional aggregate used. Composite with such a quantity of waste retained the characteristics of cement concrete, which qualified its use for construction concrete.

## 1. Introduction

One of the most important issues of current science is the problem of rational waste management. Despite the fact that the recycled use of many exploited products is possible from a technical point of view, many methods and proposed solutions encounter economic limitations. In cases where raw materials for the production of new products are easily available and their acquisition is not expensive, recycling from the point of view of enterprise is unnecessary. If the recycled materials cannot be financially justified, they are not used. In many cases, manufacturing companies reserve the possibility of using worn-out products in new production provided that they are delivered for free. This is a sign of willingness to implement economically justified recycling. In this case, the financing of collection, segregation and transport becomes such an expensive undertaking that ultimately various types of waste, the recycling of which is technically possible, end up in illegal garbage dumps. An example of the situation described above is the recycling of selected glass waste. In the case of packaging glass, activities such as collection, selection, cleaning and transport of waste to glass-works are economically justified. Due to the large scale of packaging turnover and the possibility of obtaining payment for recyclable material, technical possibilities are appropriately used here. The situation of glass waste is different, the quantities of which are small and the adjustment processes of which are complicated. Products of this type include glass lighting materials such as light bulbs and fluorescent lamps. 

A fluorescent lamp is a discharge lamp in which light is emitted by a phosphor excited by ultraviolet radiation. It most often has the shape of a pipe, covered on the inside by the mentioned phosphor filled with mercury vapor and argon, in which the source of illumination is visible radiation emitted by the phosphor layer covering the inner surface of the tube. Due to the presence of mercury vapor, the utilization of lighting elements is carried out in a way that requires many treatments in which harmful substances are neutralized. The process of utilizing fluorescent lamps is carried out under negative pressure so that mercury vapors do not get into the atmosphere. First, the used fluorescent lamps are put in the feeder, which is tightly closed, and then its contents go to the chamber, in which the contents of the feeder are heated above 200 °C. As a result of heating, evaporation of mercury occurs, while glass and aluminum tips remain in the chamber on the bottom. After filling the chamber with glass and aluminum, it is emptied, and its contents are directed to the separator. Floating mercury vapor is directed to the next chamber, which has a temperature of −75 °C, which causes mercury to pass from its gaseous to liquid state. Separation of mercury leads to it being able to be reused. This process ensures that no mercury vapor remains in the glass material that could cause health problems. However, the glass is not yet devoid of steel elements. It goes into a separator in which parts other than steel are separated after fragmentation. Phosphor is also a problematic substance that is covered with glass elements. Its removal in the process of recovery of pure glass material is also a very costly procedure.

As it can be seen in the above description the recovery of glass material is difficult and expensive, and the transport of small amounts to glass-works becomes completely economically unjustified. In such cases, it is necessary to look for other more local methods of recycling waste material. The solution proposed here is to include recycled glass in a different industry, such as the production of concrete. The possibility of including this waste in the local production of concretes would have mainly ecological benefits—the amount of matter in landfills would be reduced as well as the use of natural aggregates.

The use of glass as an additive in cementitious composites is an issue analyzed by many research teams, e.g., [1,2]. Based on the form in which the glass is introduced into the analysis mix, there are several directions of research. The oldest of the proposed solutions is the use of glass in the form of fibers [3,4,5,6,7,8,9,10,11,12]. An addition of this type positively affects the parameters of the concrete obtained. Concretes with added fibers have higher strength parameters, and are more resistant to environmental factors such as humidity, sun exposure or thawing and freezing processes. Such a beneficial effect of glass fibers on concrete has caused many companies to produce glass fibers from waste material. However, this activity is not conducted locally, and requires large energy expenditures to melt the recyclate.

Another proposed solution is the introduction of glass into concretes in the form of dust [13,14,15,16,17,18,19,20,21,22,23,24,25]. This type of additive also has a positive effect on cementitious composites. Ground waste results in better workability of the concrete, and after bonding the composite has higher compressive and tensile strength than composites made without its participation.

A relatively new idea is the introduction of glass waste into cement mixtures as a substitute for coarse aggregates. Research carried out both on glass [26,27,28,29,30,31,32,33] and similar ceramic waste [34,35,36] show that such activity is possible. The preparation of this type of aggregate is the most advantageous from an economic point of view. It does not require large amounts of energy for penetration, as happens in the case of fibers, or crushing, as in the case with dust. The fragmentation of glass into the form of coarse grains is a simple activity that does not require large energy expenditures. At the same time, it is possible to implement in standard crushers, which are popular in concrete plants.

Despite the fact that the recycling of glass waste in concrete composites is analyzed by many research teams, analyses carried out on recyclate from depleted fluorescent lamps in cement composites were not encountered. The only work devoted to this waste is [37], where this recyclate is used for the production of resin composites. Some authors have reported the problem of Alkali-Silica Reaction while studying the use of concrete with glass aggregates in the past [38,39,40]. This problem is not studied in this work. 

## 2. Materials Used in the Study

**Figure 1:**
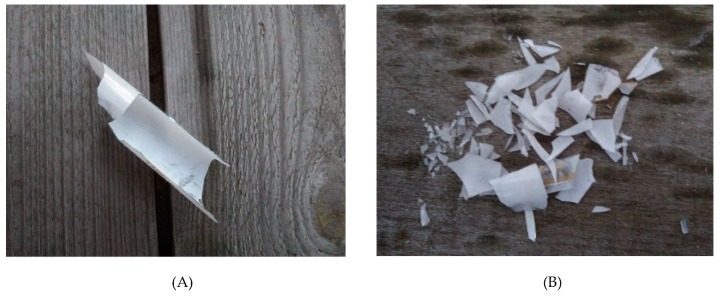
Waste from glass (**A**) glass separated from lighting equipment; (**B**) aggregate made from waste.

The research material was obtained from a company dealing in the utilization of electric waste. Exploited bulbs and fluorescent lamps were deprived of harmful substances and equipment there, which was damaged during the process. The material ultimately collected for testing was mainly broken glass tubes. The pipes were transferred to a plastic container and transported to the laboratory. Crushed glass was made using a drum crusher. The crushing of the material was carried out until all aggregate grains were less than 8 mm. The research material is presented in Figure 1.

**Figure 2:**
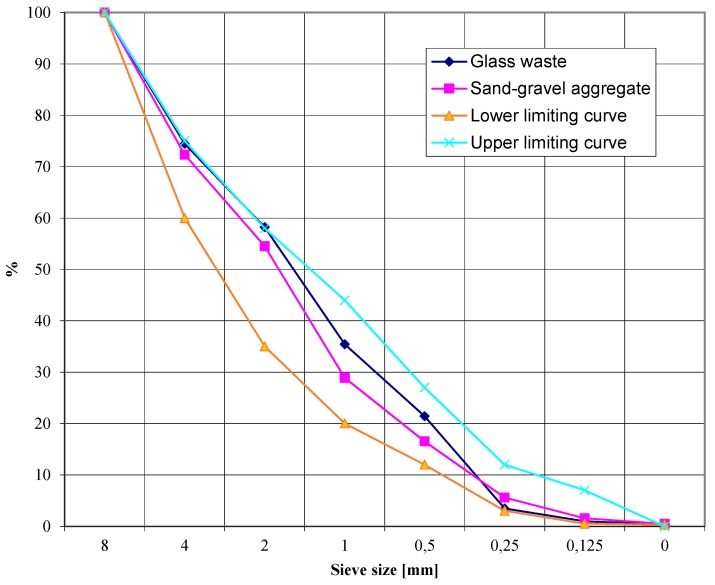
The sifting curves for glass aggregate and sand–gravel mixture against the background of boundary curves.

The aggregate obtained in this way was subjected to an analysis of basic properties [37,41,42,43,44], which is typically carried out for aggregates for cement concrete. The same tests were carried out on traditional sand and gravel aggregates. The first test performed for both types of aggregates was the assessment of the grain composition made according to EN 933-1: 2012 [41] by the sifting method. Figure 2 shows the sifting curves for glass aggregate and sand and gravel aggregate in the background of limiting curves. The sand–gravel mixture consisted of two grades: 0–2 mm and 2–8 mm, and its composition was determined by calculation method in which a sand point of 54% was assumed, which corresponds to suggestions in literature [37] for a mixture of grain size up to 8 mm.

To determine the specific density of aggregates, the standard method according to PN-EN1097-7: 2011 [44] was used. The apparent density and absorbability of the aggregate were tested using the standard method according to PN-EN 1097-6: 2011 [43]. The strength test that was carried out for the aggregates was the test of crush strength for crushing aggregates. The test was carried out in accordance with PN-B-06714-40: 1978 [45] on aggregate with a grain sizes of 0–2 and 4–8 mm. The testing stand is equipped with a container for measuring a portion of aggregate, a laboratory scale and a screen with a mesh of 1 mm. The strength test was performed on a hydraulic press using a special vessel with a piston for crushing the aggregate. The crushing index was set as a percentage share of grains, which after crushing passed through a sieve of 1 mm mesh. A comparison of results obtained for the tested aggregates, both glass and sand–gravel mixture, is given in Table 1. The table also lists the other parameters of the aggregates described, which were taken from [37].

The properties of materials such as glass and gravel, presented in Table 1, prove that the glass material itself has very high strength parameters. Glass is a homogeneous material with a crystalline structure. Gravels contain rock crumbs of various origins. A block made of glass has higher strength parameters than a block made, e.g., of low-strength weathered rocks, e.g., limestone. Despite the presented features of traditional materials and glass themselves, the authors prove that this feature is not the only one that affects the quality of the resulting composite. In this case, the decisive element turned out to be the shape of the grains used. The grain’s flatness made it impossible for the grains to form tightly in the composite. This was also the reason for the occurrence of a large amount of free spaces filled with air. The above elements significantly reduced the compressive strength of the composite. 

Gravel aggregate graining resulted in a tight arrangement of smaller sized grains between larger sized grains. This guaranteed the tightness of the crumb stack, which when bonded with leaven carried the load. In the case of glass aggregates, due to difficulties in their mutual arrangement between each other, the tightness of the crumb stack could not be maintained. Probably this fact was also the cause of the occurrence of air pores and lower strength parameters of composites with recyclate.

The comparative method [37] was used to assess the shape of the grains of the aggregate with the recycled glass. The length, width and thickness of representative grains whose occurrence was most numerous were measured. On the basis of comparison of length and width, which were three times larger than thickness, it was estimated that they were flat grains. The continuation of this study was the study of grain flatness index and the addition of statistical measures to the description of glass aggregate grains, such as the average value and standard deviation of the shape coefficients (a/b, a/c). As a result, the shape, which was crucial for the strength properties of the composites, was available to other researchers quantitatively selected representative grains of glass aggregate were subjected to dimensional testing. The smallest dimension was designated with the value of a, the larger—b, and the largest—c. The average value tested for 100 pieces of measured grains of a/b coefficients was 0.34, and the standard deviation for this value was 0.19. For the a/c ratio tested, these values were, respectively: average 0.2, and deviation 0.05.

The assessment of the type of grain texture of aggregates from waste glass materials was made on the basis of [37]. The structure was rated as smooth. Portland cement CEM I 42,5N-SR 3/NA was used for prepared concretes. It is characterized by stable physicochemical parameters of appropriate bonding time, high early and final strength parameters, low alkali content, and high resistance to aggressive chemical agents, which is popularly used is in the production of concrete mixes. The detailed values of the physicochemical parameters of the cement are given in Table 2.

As a plasticizing admixture, CHRYSO Omega 132 was used. The admixture is manufactured using the latest hybrid polymer technology. This technology uses knowledge about the synthesis of molecules, and it enables a strong reduction of the amount of mixing water, long-term maintenance of the consistency of the concrete mix, and the homogeneity and cohesion of the concrete mix. The basic technical parameters of the admixture from its technological sheet are presented in Table 3.

Fly ash was used as a concrete additive. It was a fine-grained dust consisting mainly of spherical, grained grains, obtained during the combustion of bituminous coal with the participation of co-combustion materials. The basic components of the ash were aluminosilicates (SiO_2_ and Al_2_O_3_). The technical data sheet of the product ensured that it complies with the requirements of the EN 450-1 standard as class A in terms of roasting loss and N category in the fineness range and has pozzolanic properties. The basic characteristics of fly ash from its technical sheet are given in Table 4.

All components used in the study (including fly ash) are popularly used in Poland in the industrial production of concrete. CONTR concrete base mix presented in Table 5 with the content of gravel aggregate alone was the same as that produced for construction purposes. The only variable parameter in the research was to be the use of recycled aggregates as a substitute for traditional aggregate.

## 3. Mix Design Method

The theoretical and experimental method was used to determine the prescription of the concrete mix [37,46]. Due to the similarity of the granulation of the recycle aggregate and the comparative mixture, the design process was carried out for the concrete containing only sand and gravel aggregate. During the design of the concrete mixture recipe, the C 25/30 strength class was established. Using the glass waste aggregate as a substitute for sand and gravel mixture in various proportions, the following series of mixtures were made: a control mixture (CONTR) containing all sand and gravel aggregate, CG-10 containing 10% glass cullet, CG-30 containing 30% glass cullet, CG-50 containing 50% glass cullet and CG-100 containing 100% glass cullet.

The design of the concrete mixture began with determining the qualitative components and determining their basic properties. Based on the technology sheets of the supplied substrates and own tests, the volume densities of the concrete constituents, natural aggregate moisture, aggregate sand points and a sand point of 54% were determined. On the basis of general formulas [37], the proportions of aggregates and then the number of individual concrete components were selected. The final composition of CONTR concrete is presented in Table 5, and the composition of all mixtures made is presented in Table 6.

## 4. Results and Analysis

All prepared concrete mixtures were subjected to consistency tests according to EN 12350-2—Concrete mix tests, Part 2—Slump test [47]. During all tests on the consistency of concrete, the concrete mix was laid in a cone in three layers, and in each case compacted with a special rod 25 times. The measurement was made six times on the basis of the reading of the difference in the height of the conical form, and the upper surface of the concrete mix, which fell after the cone was removed. The results of measurements of all concrete mixes are given in Table 7.

All types of concrete were made on the basis of the same working recipe, replacing only the mixture of sand and gravel aggregates with glass aggregate. The dosage of water, chemical admixture, and additives were implemented in the same amounts. The observed precipitation results of the cone were different for different mixtures, the overall liquidity of the mixture increasing with the amount of glass additive. It is assumed that the glass, as a non-absorbent material, did not absorb water, which caused it to become plastic. This feature testified to the positive effect of the glass material on the concrete. Achieving the assumed consistency when dosing a smaller amount of mixing water positively affects the W/C index and improves all concrete properties. During the study, attention was also paid to the increasing variability index along with the addition of glass. This phenomenon proved that the addition of glass waste meant that the mixture was less homogeneous, and this feature is unfavorable from the point of view of concrete production.

Another test that was carried out for all fresh concrete mixtures was the air content test [37] using the Boyle-Mariotte method. The TESTLAB 5l porosimeter was used for the study. The results of this study are presented in Table 8.

Analysis of the results of an air content test in a concrete mix proved that with the same components, the air content in the mixture increased significantly. This was an unexpected phenomenon because the quantities of all components were the same. Also, aeration agents were not added to the mixture preparation. Two hypotheses were put forward in order to explain this phenomenon. Firstly, assuming that the glass is a neutral material that does not react chemically with the components of the concrete, it was assumed to react with the components of the concrete that the phosphor layer could be coated with fluorescent tubes. As a result of the chemical reaction, a gas could appear that caused differences in the results of the air content test. The second hypothesis was that air gaps were formed between the irregular shaped particles, which could not leave the mixture during the test. The explanation and confirmation of hypotheses was planned to be carried out during microscopic examination.

The next study was a study of the volume density of concrete samples. The volume density was tested on 10 × 10 × 10 cm cubic samples. Six samples of each concrete were prepared. The test was carried out in accordance with EN 12390-7 [48]. The results of the study are presented in Table 9.

The mean bulk densities measured for the concrete tested were, respectively, CONTR-2300 kg/m^3^, CG10–2290 kg/m^3^, CG30-2180 kg/m^3^, CG50-1960 kg/m^3^, and CG100-1660 kg/m^3^. The results of this study show that this property was different for all concrete types. The density of concrete was lower, the more glass aggregate was used. The lower density of the crushed aggregate itself could have an impact on this feature. Probably, however, the differences in density were largely caused by the increasing air content in the mixture.

Water absorption was tested on identical samples as volumetric density. Six samples of each concrete were also tested. The samples were immersed in water and they remained there until their mass stabilized. The absorbability was calculated according to [37] as the ratio of the amount of water the concrete was able to absorb relative to the dry concrete mass expressed as a percentage. The results of the study are presented in Table 10.

The water absorption varied, and for the mixtures CONR, CG10, CG30 CG50 and CG100 was 3.52%, 3.73%, 4.73%, 5.89% and 8.93%, respectively. This proved, therefore, that with the increasing amount of glass aggregate, the absorption of concrete increased, which probably also caused the occurrence of air voids in the concrete volume. Analyzing the results of this study, attention was also paid to the volatility index increasing along with the content of glass waste. This was another proof that the samples with the increasing amount of waste are more heterogeneous.

Another test was the tensile (flexular) strength test of hardened concrete. As the largest dimension of the aggregate used to make the concrete, the 8 mm dimension for the strength tests was prepared in the form of bars measuring 4 × 4 × 16 cm. After forming, the samples were subjected to moisturizing care in a closed container partially filled with water. After the third day after the molding, the samples were removed from the molds and then they were still in the described environment for the following days. The tests were carried out in accordance with EN 12390-3 [49]. For this type of examination, 9 samples for each type of concrete were made. Tensile (flexular) strength tests were carried out in the laboratory on the CZ250 kN testing machine from Controls. The results are presented in Table 11.

Analysis of the test results has shown that samples with a higher content of glass waste had lower tensile strength. Comparing the tested values for CONTR concrete strength, the decrease in strength was found for concretes: CG-10-17%, CG-30-10.5%, CG-50-45.2% and CG-100-53.8%. In the obtained results, similarly to in the case of previous studies, attention was drawn to the very high coefficient of variation obtained especially for samples with the addition of 100% glass aggregate. 

**Figure 3:**
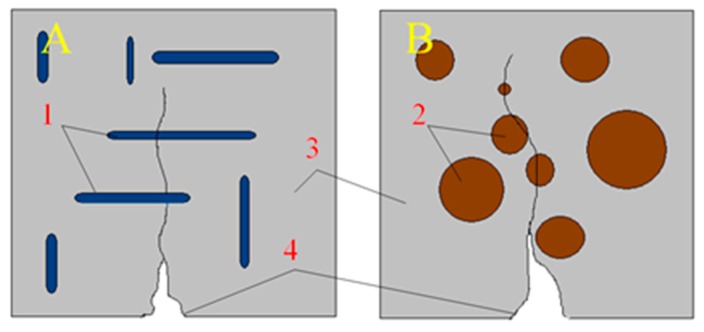
Scheme of the crack propagation and destruction of the middle part of the samples during the bending test of the composite: (**A**) crack propagation in a composite containing flat grains; (**B**) crack propagation in a composite containing round grains. Marks: 1—flat glass grains, 2—gravel grains, 3—binder resin, 4—propagating the crack. Flat grains located perpendicular to the propagating scratches are a kind of dispersed reinforcement improving the bending strength of the composite. In this process they are subject to stretching and protect against further crack propagation.

Interesting was also the increase in tensile strength for a composite containing 30% recyclate. With reference to [37], it can be assumed that a small percentage of the content of glass grains may constitute reinforcement in the composite and improve this technical feature. Referring to the above-mentioned work, an exemplary scheme of this phenomenon is presented in Figure 3.

Another test was the test of crushing strength of hardened concrete. The halves of the samples obtained during the tensile (flexular) strength test were applied. The test was carried out on the same testing machine as the previous one. The results are presented in Table 12.

**Figure 4:**
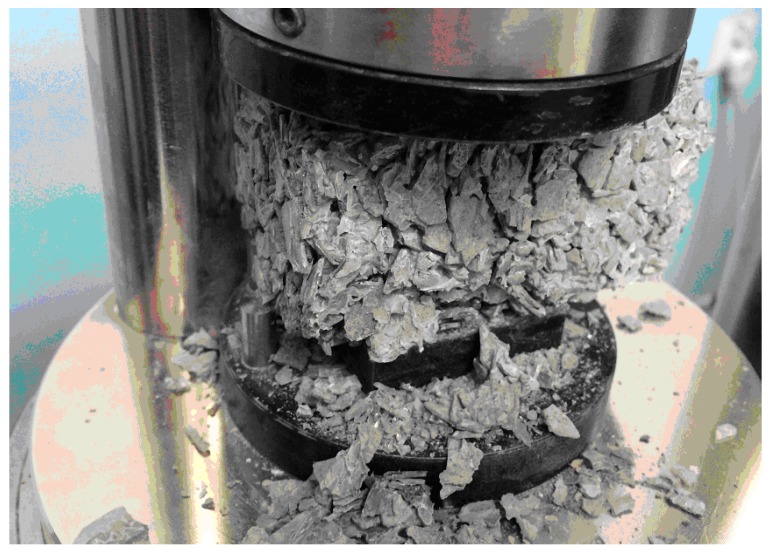
View of the CG-100 concrete sample during the compressive strength test.

The most favorable compressive strength test results were observed for CONTR concrete, i.e., without glass content in its composition. The worst for the series were observed when the glass content of fluorescent lamps in the aggregate was 100%. Analyzing the results again, it was found that the addition of glass negatively affects the characteristics of concrete, and it is a linear relationship, i.e., that the higher the glass content from exemplary glass in the aggregate, the lower the compressive strength. It should be emphasized that the difference in extreme values was as much as 10-fold. The negative impact of glass from fluorescent lamps on the final compressive strength of concrete is visible. Referring to the concrete strengths of subsequent series, i.e., CG-10, CG-30, CG-50 and CG-100, CONTR concrete strength was estimated to mean that the percentage decrease in strength was 13.5%, 32.5%, 69.3%, and 90.8%, respectively. During the tests, some anomalies were also noted. Samples with the glass content were more damaged by peeling than cracking. A sample of CG-100 concrete during the compressive strength test is shown in Figure 4. This study also proved a concrete composite containing 30% recyclate retains a relatively high compressive strength—higher than 30 MPa—which classifies it as structural concrete.

## 5. Microscopic Analysis

**Figure 5:**
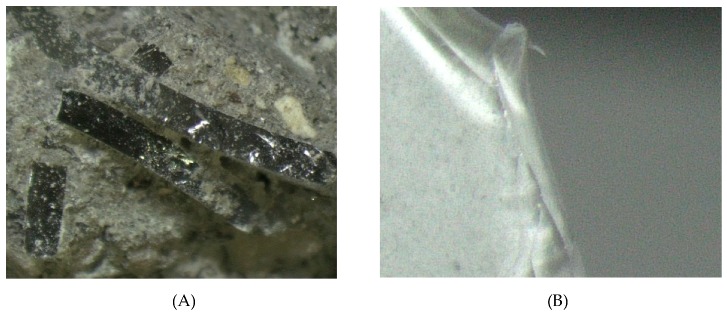
Enlarged glass aggregate grain (**A**) in the leaven, and (**B**) in proximity to the surface structure.

The last stage of the conducted research work was microscopic analysis, carried out using an optical microscope. These analyses were conducted in order to clarify the phenomenon of lowering the quality of concretes due to the addition of lighting glass. The glass material itself, tested by methods such as traditional cracks, showed advantageous features as aggregate. Microscopic analyses were planned to confirm one of the hypotheses regarding the increasing amount of air in concrete resulting from the addition of glassware. The first stage of microscopic analysis was the observation of phosphor-coated glass surfaces. Fragments of concrete samples after destruction became detached during the test. Figure 5A,B shows an enlarged glass aggregate grain in a leaven and in a close relationship to the surface structure, respectively. 

**Figure 6:**
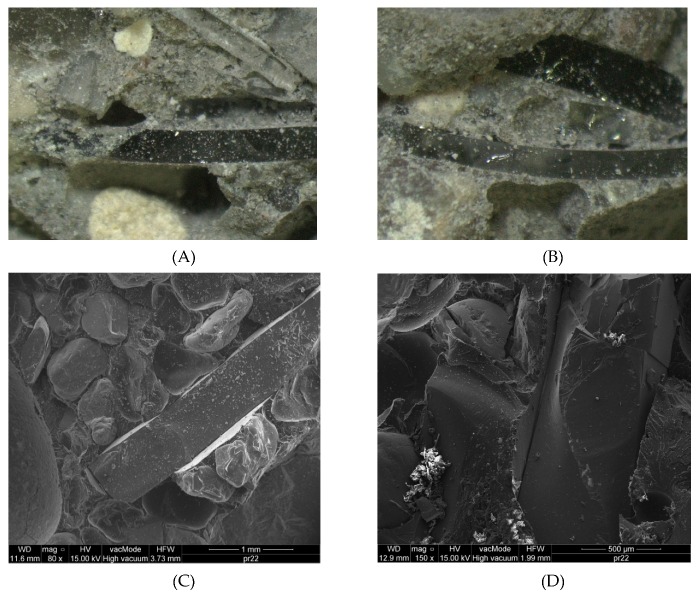
Two examples of grains of glass aggregate in leaven and air voids located in their close vicinity. Magnification: (**A**,**B**) 10x; (**C**) 80x; (**D**) 150x.

Microscopic analysis showed that the luminous phosphor remained on the surface of the glass elements. This was proof that there was no chemical reaction with the cement components. Subsequent microscopic analyses were carried out on the cracked edges of the samples that were formed after testing the tensile strength (flexular) of concrete. Exemplary samples are shown in Figure 6.

**Figure 7:**
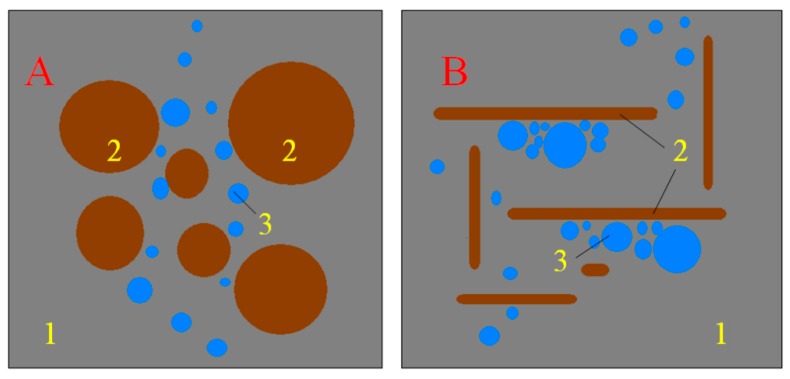
Scheme of air retention in a concrete mix during vibration. (**A**) Mixture with aggregate shaped, (**B**) mixture with flat aggregate. Markings in the drawing: 1—cement paste, 2—aggregate grains, 3—air bubbles moving upwards during vibration.

In Figure 6, you can see air voids near the grains of the glass aggregate. The flat shape of the glass aggregate was probably the cause of the accumulation of air under its grains. In the case of round grains, which occur in predominance in traditional aggregates, air bubbles are able to leave the mixture freely during vibrations. In the case of glass aggregate, the air does not have this possibility. This problem is illustrated in Figure 7.

This fact explains both the unfavorable parameters of the tested concretes (compressive strength of the glass itself and its low water absorption) and the linear relationship between deterioration of properties and the content of glass aggregate. As this phenomenon cannot be controlled and depends mainly on the way in which the grains are arranged in a mixture of concretes with a high content of glass aggregates, they have a large heterogeneity. This was confirmed by high variability indices of the examined properties.

## 6. Summary and Conclusions

Despite the obtained results confirming the general adverse effect of ceramic glass aggregates on the performance parameters of cement composites, the proposed solution for implementation in industrial activities is recommended. Special attention is paid here to the fact that substituting 30% of aggregate for recyclable aggregate makes it possible to obtain a concrete composite with high technical parameters. Such a composite has a compressive strength of 31.7 MPa and a tensile (flexular) strength of 7.43 MPa, as well as other positive properties that qualify it for the constructional composite. This action is recommended especially for pro ecological reasons. Thanks to such solutions, local glass waste can be used for traditional concrete production, and this has a doubly positive environmental effect: unwanted material is utilized, and at the same time, the consumption of natural aggregates is reduced.

Among the specific applications, it should be particularly emphasized that:

1. It is possible to obtain glass material in the form of crushed glass grains from which the concrete aggregate can be made from worn-out lighting materials.

2. Aggregate formed from the crushing of glass waste examined by methods for traditional aggregates has favorable parameters—no worse than those tested for traditional aggregates such as sand and gravel.

3. The concretes made with the use of glass aggregate have less advantageous characteristics with greater amounts of glass aggregate being used in their production. The reason for the deterioration of the features is the flat shape of the grains of the recycled aggregate derived from lighting waste. Flat grains cause air bubbles during vibrations to accumulate under them, creating voids, and the high content of air voids formed under the grains of the flat aggregate are the cause of the deterioration of concrete features and their high heterogeneity.

4. A composite containing 30% glass aggregate has technical features that qualify it as a construction material. The use of glass recyclates for concrete composites in the amount of 30% of aggregate mass gives a satisfactory effect and is in line with environmental trends. Higher percentages are generally less recommended, as the occurrence of air voids will unavoidably grow. To some extent, this can be overcome by increasing the fluidity of the mix, extending the vibration time for the automatic arrangement of close grains, or using less liquid mix and introducing vibro pressing.

5. This research raised some safety concerns. The aggregate used in the tests has numerous sharp edges and may cause injury when in contact with human skin. This fact emphasizes how difficult this waste is in respect to its utilization and disposal. Technological pollution by phosphor does not allow direct use of waste in the production of new glass. Using it in crushed form, e.g., as aggregate for hardening embankments, similarly to processes that use concrete or ceramic destruct, is also not possible. Tests of this kind caused injury to car tires. On the other hand, waste disposal by landfill is unfavorable from an environmental point of view. It is a devastation of the material and energy that were used in the production process of products. All the above aspects prompted the authors of the article to attempt to use this material for the production of composites in which it would be a substitute for natural aggregates. One of the authors of the article has extensive engineering experience in the production of concrete composites and their use in industrial conditions. These experiences show that both machine operators at concrete mixing plants in the production of concrete and employees laying the composite in formwork (also the vibration process) do not have direct contact with the cement composite. In these processes, employees’ skin is not allowed to come into contact with the concrete mix—also due to the adverse effects of the cement paste itself on human skin. In specific cases, e.g., in the event of a breakdown or experimental work, special precautions should be taken, and the possibility of injury should be taken into account. Also, as described in the introduction, the initial treatment of glass must ensure that no mercury vapor remains in the glass material that could cause health problems.

## Figures and Tables

**Table 1:** Basic parameters of aggregates used for concretes.

Type of Aggregate/Property	Unit	Traditional Aggregate: Sand, Gravel	Aggregate with Glass Lighting Waste
Specific density	kg/m^3^	2650	2630
Volumetric density	kg/m^3^	2200	2620
Compressive strength	MPa	33	650
Absorbability	%	2.1	0.1
Crushing index	%	14.3	34.5

**Table 2:** Physical and chemical parameters of cement CEM I 42,5N–SR 3/NA.

Property	Unit	Average Result	Requirement
Bonding strength (early)	min	233	>60
Bonding strength (final)	min	291	
Water demand	%	27.5	
Stability of volume	mm	1.1	<10
Specific surface	cm^2^/g	3688	
Compressive strength: after 2 days	MPa	23.9	<10
Compressive strength: after 28 days	MPa	55.9	> 42.5 < 62.5
Chemical analysis: SO_3_	%	2.77	<3.0
Chemical analysis: Cl	%	0.070	<0.10
Chemical analysis: Na_2_Oeq	%	0.53	<0.6

**Table 3:** Basic properties of plasticizing admixture.

Property	Description
Form	Uniform liquid
Color	Brown
Density (20 °C):	1.075 +/− 0.02 kg/dm^3^
pH:	5 +/− 1
Ions Cl^−^:	Up to 0.1%
Alkali to Na_2_O ratio	Up to 2.0%

**Table 4:** Basic properties of fly ash.

Parameter	Unit	Value	Assessment Method
Form	-	Fine grain powder	visual
Color	-	grey	visual
Smell	-	none	-
Density	kg/cm^3^	2.05	EN 1097-6
Bulk density	kg/cm^3^	1.1	EN 1097-3
Alkalinity	pH	Less than 11.5	PN-EN-ISO 10523

**Table 5:** Composition of the control mixture.

No.	Substrate	Quantity in kg/m^3^	Density kg/dm^3^	Volume dm^3^	% Mass
1	Cement CEM I 42,5N–SR 3/NA	250	3.10	80.65	10.49
2	Fly ash	40	2.05	19.51	1.68
3	Grains 0–2 mm	1034	2.65	390.19	43.40
4	Grains 2–8 mm	881	2.65	332.38	36.97
5	Admixture	2.5	1.07	2.34	0.10
6	Water	175	1.00	175.00	7.35
	Sum	2382.31		1000.07	100.00

**Table 6:** Compositions of all prepared concrete mixtures.

No.	Substrate	CONTR	CG-10	CG-30	CG-50	CG-100
1	Cement CEM I 42,5N – SR 3/NA	250	250	250	250	250
2	Fly ash	40	40	40	40	40
3	Grains 0–2 mm	1034	931	724	517	0
4	Grains 2–8 mm	881	793	617	441	0
5	Admixture	3	3	3	3	3
6	Water	175	175	175	175	175
7	Aggregate with glass waste	0	192	575	958	1915

**Table 7:** Results of testing the consistency of concrete mixes.

Mixture	Sample Number	Cone Descent [mm]	Average Descent [mm]	Standard Deviation [mm]	Variability Index [%]	Consistency Class
CONTR	1.1–1.6	120–128	124.67	1.78	1.43	S3
CG10	2.1–2.6	135–146	141.17	3.11	2.20	S3
CG30	3.1–3.6	158–183	167.50	6.83	4.08	S4
CG50	4.1–4.6	165–208	191.00	13.00	6.81	S4
CG100	5.1–5.6	186–219	199.67	15.67	7.85	S4

**Table 8:** Results of testing the air content of concrete mixes.

Mixture	Sample Number	Air Content [%]	Average Air Content [%]	Standard Deviation [mm]	Variability Index [%]
CONTR	1.1–1.6	1.7–1.8	1.75	0.05	2.86
CG10	2.1–2.6	1.9–2.0	1.92	0.03	1.45
CG30	3.1–3.6	2.4–2.6	2.48	0.06	2.24
CG50	4.1–4.6	6.5–8.2	7.23	0.43	5.99
CG100	5.1–5.6	9–11	10.17	0.61	6.01

**Table 9:** Results of volumetric density testing of prepared concretes.

Mixture	Sample Number	Volumetric Density [g/cm^3^]	Average Volumetric Density [g/cm^3^]	Standard Deviation [g/cm^3^]	Variability Index [%]
CONTR	1.1–1.6	2.285–2.32	2.30	0.01	0.60
CG10	2.1–2.6	2.27–2.297	2.29	0.01	0.40
CG30	3.1–3.6	2.172–2.215	2.18	0.03	1.16
CG50	4.1–4.6	1.91–1.992	1.96	0.03	1.42
CG100	5.1–5.6	1.609–1.727	1.66	0.04	2.10

**Table 10:** Absorption test results.

Mixture	Sample Number	Absorbability [%]	Average Absorbability [%]	Standard Deviation [%]	Variability Index [%]
CONTR	1.1–1.6	3.21–3.76	3.52	0.20	5.62
CG10	2.1–2.6	3.4–4.13	3.73	0.22	5.84
CG30	3.1–3.6	4.57–4.94	4.73	0.13	2.68
CG50	4.1–4.6	5.49–6.54	5.89	0.23	3.93
CG100	5.1–5.6	7.67–10.65	8.93	0.76	8.50

**Table 11:** Results of tensile (flexular) testing of concrete samples.

Mixture	Sample Number	Tensile Strength MPa	Average Tensile Strength, MPa	Standard Deviation, MPa	Variability Index %
CONTR	1.1–1.9	5.81–10.19	8.30	0.78	9.36
CG10	2.1–2.9	5.21–7.91	6.90	0.69	10.02
CG30	3.1–3.9	6.08–8.23	7.43	0.39	5.19
CG50	4.1–4.9	3.6–5.67	4.55	0.63	13.79
CG100	5.1–5.9	2.51–5.17	3.84	0.73	19.02

**Table 12:** Results of compressive strength tests for concretes.

Mixture	Sample Number	Compressive Strength MPa	Average Compressive Strength, MPa	Standard Deviation, MPa	Variability Index %
CONTR	1.1–1.9	36.95–52.75	46.19	3.23	7.00
CG10	2.1–2.9	30.27–47.13	39.95	4.38	10.97
CG30	3.1–3.9	22.59–39.54	31.17	4.58	14.68
CG50	4.1–4.9	8.72–20.36	14.16	2.45	17.29
CG100	5.1–5.9	2.33–5.46	4.23	0.93	21.98
